# Optimization strategy of Co_3_O_4_ nanoparticles in biomethane production from seaweeds and its potential role in direct electron transfer and reactive oxygen species formation

**DOI:** 10.1038/s41598-024-55563-y

**Published:** 2024-03-01

**Authors:** Mohamed A. Hassaan, Marwa R. Elkatory, Mohamed A. El-Nemr, Safaa Ragab, Ahmed El Nemr

**Affiliations:** 1 Environment Division, National Institute of Oceanography and Fisheries (NIOF), Kayet Bey, Elanfoushy, Alexandria, 21556 Egypt; 2 Advanced Technology and New Materials Research Institute, SRTA-City, New Borg El-Arab City, Alexandria, 21934 Egypt; 3 Department of Chemical Engineering, Faculty of Engineering, Minia University, Minia, 61519 Egypt

**Keywords:** Co_3_O_4_NPs, Biogas, *C. myrica*, Direct electron transfer, Biomethane, Reactive oxygen species, Chemical engineering, Photocatalysis, Energy

## Abstract

In the present study, three process parameters optimization were assessed as controlling factors for the biogas and biomethane generation from brown algae *Cystoceira myrica* as the substrate using RSM for the first time. The biomass amount, Co_3_O_4_NPs dosage, and digestion time were assessed and optimized by RSM using Box-Behnken design (BBD) to determine their optimum level. BET, FTIR, TGA, XRD, SEM, XPS, and TEM were applied to illustrate the Co_3_O_4_NPs. FTIR and XRD analysis established the formation of Co_3_O_4_NPs. The kinetic investigation confirmed that the modified model of Gompertz fit the research results satisfactorily, with *R*^2^ ranging between 0.989–0.998 and 0.879–0.979 for biogas and biomethane production, respectively. The results recommended that adding Co_3_O_4_NPs at doses of 5 mg/L to *C. myrica* (1.5 g) significantly increases biogas yield (462 mL/g VS) compared to all other treatments. The maximum biomethane generation (96.85 mL/g VS) was obtained with *C. myrica* at (0 mg/L) of Co_3_O_4_NPs. The impacts of Co_3_O_4_NPs dosages on biomethane production, direct electron transfer (DIET) and reactive oxygen species (ROS) were also investigated in detail. The techno-economic study results demonstrate the financial benefits of this strategy for the biogas with the greatest net energy content, which was 2.82 kWh with a net profit of 0.60 USD/m^3^ of the substrate and was produced using Co_3_O_4_NPs (5 mg/L).

## Introduction

One of the biggest problems facing the world in recent years is managing waste and energy needs. Rapid population growth has caused serious problems in terms of energy needs and disposal of waste^1–7^. Anaerobic digestion (AD) of agricultural wastes is the most effective method of addressing these issues because it allows for the stabilization of wastes and recovery of biomass energy^8–11^. Organically rich-biomass is a possible source for sustainable methane generation^2^. Algae grow more quickly and produce a better area than traditional crops, particularly in the summer^1,2^. The utilisation of this waste as biomass for the generation of biomethane is an appealing waste recycling method when considering environmental protection and energy recovery. Due to its low degree of hydrolysis and degradability, algal biomass has a poor rate of conversion into energy materials^10,11^. As a result, several treatment techniques, including mechanical, thermal treatment, and chemical, are created to aid in the destruction of algae. However, these techniques are constrained by high chemical and energy requirements as well as challenging operations^10^. With industrial uses of nanoparticles (NPs) in catalysts, coatings, food, drugs, sensors, paints, clothing, agriculture, antimicrobials, and packaging, there has been a dramatic change in science and technology^12^. Organic waste anaerobic digestion (AD) has been employed to promote biological activity using NPs. Biogas, which is comprised of methane (CH_4_) (55–70%), carbon dioxide (30–45%), and lower amounts of hydrogen sulphide (H_2_S) (0–30,000 ppm), is created when waste is digested^13^. As opposed to their bulk forms, nanomaterial nanoparticles (NPs) provide several benefits and features that have led to their widespread usage in a diversity of applications^2,3^. Former batch investigations have demonstrated that adding NPs (Fe, Fe_3_O_4_, Co, and Ni) to an AD reactor handling manure enhanced CH_4_ generation by 46–117%^14^. This is because adding NPs can enhance organic decomposition^15^. In addition to substantially promoting microbial growth, Fe3O4-NPs were preferred due to their low toxicity, ability to increase biogas and CH_4_ generation, and low toxicity. Fe_3_O_4_ and ZnO NPs were not commercially viable for use^7,16^.

Through methanogenesis in the AD procedure, metallic NPs (Cu, Fe, Co, Ni, Mn, and Zn) induce DIET to boost CH_4_ production^17^. Metals and non-metals are used in the DIET process as electroconductive materials for transporting electrons between the *Geobacter* and *Methanoseta* species^18^. According to Wu et al.^19^, high biogas yields from DIET and mixed microbial cultures for CH_4_ generation are stable during methanogenesis because there is enough electron transfer between the microorganisms. The most generous approach is methanogenesis, in which metabolically active methanogens assist CO_2_ in producing CH_4_ via metallic NPs. *Geobacter* species use their methanol metabolism genes in bacterial aggregates, and metallic NPs (Fe, Ni, Cu, Co, and their complexes, such as Au-SH-SiO_2_, zeolitic imidazolate framework-(ZIF), ZIF-Ni/ZIF-Fe complexes, and CuNPsZIF8) increase conductivity by facilitating electron transfer between microbes^20,21^.

In this work, the intricacies of the DIET mechanism were examined. The substantial interest in metal oxide nanoparticles has prompted the production of several nanoparticles with exceptional characteristics. Cobalt oxide nanoparticles (Co_3_O_4_NPs), which exhibit exceptional corrosion and oxidation resistance, may be used daily^22^. Co_3_O_4_NPs have recently received attention as a potential supercapacitor and battery material^23^. Additionally, Co_3_O_4_NPs have been utilized in sensors, energy storage system electrodes, and catalysis^24^. As far as we know, no study has been done on the effects of adding manufactured cobalt oxide nanoparticles (Co_3_O_4_NPs) to the brown algae *C. myrica* and cow manure on CH_4_ output and bioreactor performance. And the only published paper was on the water hyacinth Ali et al.^25^ did not explain the impact of Co_3_O_4_ on the DIET and suggested that a low dosage of Co_3_O_4_ (3 mg/L) is favorable. As a result, this study may be the first to investigate the effects of Co_3_O_4_NPs on biogas and CH_4_ generation, as well as to clarify how the dosage of Co_3_O_4_NPs affects diet and methane production. When O_2_ is introduced, various reactive oxygen species (ROS) are quickly generated. These species include hydrogen peroxide (H_2_O_2_), superoxide radicals (O_2_^•–^), and hydroxyl radicals (^•^OH), which destroy cell membranes, proteins, and DNA^26^ and have an impact on the generation of biogas and biomethane. Additionally, a detailed explanation of the suggested ROS generation mechanism following the addition of Co_3_O_4_NPs is provided. Machine learning is appropriate when dealing with situations where the link between the input and output parameters is unknown. There are obstacles to using mathematical equations, such as picking parameters, using assumptions to interpret equations, and having trouble deriving the equations^27^. Several publications are on the practical applications of Response Surface Methodology (RSM) based modelling in predicting the biogas generation in the AD system with a high *R*^2^ of more than 0.9^28^. Response surface methodology (RSM) effectively optimizes multiple parameters at the same time to eliminate the shortcomings of optimization techniques that require a large number of experiments and thus considerable time. RSM considerably decreases the number of trials required to predict the conditions for optimal performance, studies the interactive effects between multiple factors, improves the interpretation of complex phenomena and provides a basis for process scaling and maximising process efficiency^11^. One such RSM experimental design, the Box-Behnken design, is the most widely used statistical method to predict the relationship between the independent variable and results^11^. This research primarily emphasizes the statistical optimization of RSM for brown algae into biogas and biomethane production by employing Box-Bencken design through Co_3_O_4_ pretreatment and its possible involvement in DIET and ROS formation.

## Materials and methods

### Materials

Absolute alcohol, sodium hydroxide, cobalt nitrate hexahydrate (Co(NO_3_)_2_^**.**^6H_2_O), and deionized water (DW) were all employed. All chemicals utilized in the synthesis process were bought from Sigma Aldrich of the United States. The analytical grade compounds might be employed right away without further purification. *C. myrica*, a green alga, was harvested from the Mediterranean Sea off the coast of Alexandria in Egypt and gently treated with water to remove contaminants before being rinsed multiple times with distilled water and dried in an oven to produce the final product.

### ***Synthesis of Co***_***3***_***O***_***4***_***NPs***

The Co_3_O_4_ nanoparticles (Co_3_O_4_NPs) were made using the co-precipitation technique^29,30^. Deionized water (DW) was used to dissolve Co(NO_3_)_2_^**.**^6H_2_O (0.2 M), which was then magnetically agitated for 30 min. Next, NaOH (0.2 M) was added dropwise to the aforesaid solution. Centrifugation separates the light purple precipitates after the mixture is agitated for three hours at 60 °C. The precipitates are cleaned three times with DW and three times with pure alcohol. Overnight, an oven set to 80 °C dries the precipitates that have been collected. The produced Co_3_O_4_NPs are kept at 500 °C for three hours in an electric furnace.

### Characterization and measurement

The methods listed below were applied to describe the Co_3_O_4_NP samples: Fourier transform infrared (FTIR) spectroscopy VERTEX70, Germany, connected to platinum ATR unit model V-100 in the wavenumber range (400–4000 cm^–1^), X-ray diffractograms (XRD) was performed using Bruker Meas Srv (D2-208219)/D2-2082019 diffractometer that operates at 30 kV, 10 mA with Cu tube (λ = 1.54 Å) in a range from 5° to 80°. JOEL JSM 6360 LA, a scanning electron microscope, and JOEL 2100, a transmission electron microscope, were used to examine the morphological structure of Co_3_O_4_NPs. On the BELSORP Mini II, manufactured by BEL Japan, Inc. in Osaka, Japan, the mean pore width and specific surface area were calculated using Brunauer–Emmett–Teller (BET). Using the SDT650-Simultaneous Thermal Analyzer device, thermal analysis (TGA) was carried out in the range of 25–900 °C with a 5 °C per minute ramping temperature.

### Biogas and biomethane tests

Laboratory tests were conducted on reactors in associated digesters built of cylindrical syringes^5–7^. In every test, 100 mL glass syringes were used, and each one was placed with the reverse end towards the reactor lid^31,32^. As feedstock, different amounts of milled *Cystoceira myrica* (dried weight) were used with different amounts of Co_3_O_4_NPs as designed by the Box-Behnken design (BBD) (Table 1). The untreated *Cystoceira myrica* was given 30 g (wet weight) of manure in each syringe. For ten minutes, N_2_ was flushed through the working volume. Each anaerobic degradation setup underwent three repetitions. It took the inoculum five days of pre-incubation, or until there was no apparent biogas generation. The digesters were shaken continually while being incubated at 37 °C for 150 rpm. The examined *C. myrica* had an 82.60% VS content. The C/N ratio, however, is just approximately 13.82 percent. In contrast, manure has a VS content of 84.7% and a C/N ratio of around 11.6%^1^. A plastic syringe used to sample the gas and re-inject it into the garbage was equipped with a three-way valve, and it was used to quantify the volume of biogas generated each day. As for measuring methane (CH_4_) percentages, a portable gas analyzer (Geotech, GA5000, Warwickshire, UK) was used.

**Table 1:** In batch studies, an overview of the pre-treatment techniques and substrates utilised to estimate *Cystoceira myrica*'s biogas and biomethane output.

No	Sample	Ratio (g:g)*
1	*C. myrica* + manure (Control)	0.5:30
2	*C. myrica* + manure + 5 mg/L Co_3_O_4_NPs	0.5:30
3	*C. myrica* + manure + 10 mg/L Co_3_O_4_NPs	0.5:30
4	*C. myrica* + manure + 15 mg/L Co_3_O_4_NPs	0.5:30
5	*C. myrica* + manure (Control)	1:30
6	*C. myrica* + manure + 5 mg/L Co_3_O_4_NPs	1:30
7	*C. myrica* + manure + 10 mg/L Co_3_O_4_NPs	1:30
8	*C. myrica* + manure + 15 mg/L Co_3_O_4_NPs	1:30
9	*C. myrica* + manure (Control)	1.5:30
10	*C. myrica* + manure + 5 mg/L Co_3_O_4_NPs	1.5:30
11	*C. myrica* + manure + 10 mg/L Co_3_O_4_NPs	1.5:30
12	*C. myrica* + manure + 15 mg/L Co_3_O_4_NPs	1.5:30

*Ratio (g:g) = *gram of total solid (TS)of substrate to gram of wet weight of manure.*

### Study of kinetics and statistical analysis

The cumulative biogas production was calculated using the modified Gompertz model Eqs. (1)^33,34^.

1 $$ M = Pb \times \exp \left\{ { - \exp \left[ {\frac{{R_{m} \times e}}{{Pb\left( {\lambda - t} \right)}} + 1} \right]} \right\} $$

where M represents the methane yield (L/g VS added) over time *t* (days), *Pb* represents the substrate's maximum biogas capacity (L/g VS added), *t* represents the time (day), *R*_m_ represents the highest biogas rate, and *e* represents a constant equal to 2.7183. The accuracy of the studied model is based on the correlation coefficient (*R*^2^) calculated using Origin 2020b software.

### Optimization study response surface methodology (RSM)

The influence of Co_3_O_4_NPs on the generation of biogas from C. myrica is studied using the Box-Behnken design (BBD), which is calculated using the State-Ease design expert v 13.0.5.0 software. The impacts of three independent variables (InV) (A: Biomass quantity, B: Co_3_O_4_NPs dosage, and C: Contact Time) on the response (R: Biogas or biomethane yield) were examined using response surface methodology (RSM) in order to optimise the effective parameters on the adsorption process. The experiment's range and variables are listed in Table 2.

**Table 2:** The biogas and biomethane study's range and levels.

InV	Code	Units	Lowest	Highest	Mean	STD
Biomass amount	A X1	g	0.5	1.50	1.00	0.3536
Co_3_O_4_NPs dose	B X2	mg/L	5.00	15.00	10.00	3.54
Contact time	C X3	day	11.00	33.00	22.00	7.78

ANOVA (analysis of variance) was applied to test the resulting model statistically. Surface contour plots were applied to test the relationships between the variables.

## Results and discussion

### Characterization of Co_3_O_4_NPs

#### Fourier transform infrared spectra (FTIR)

**Figure 1:**
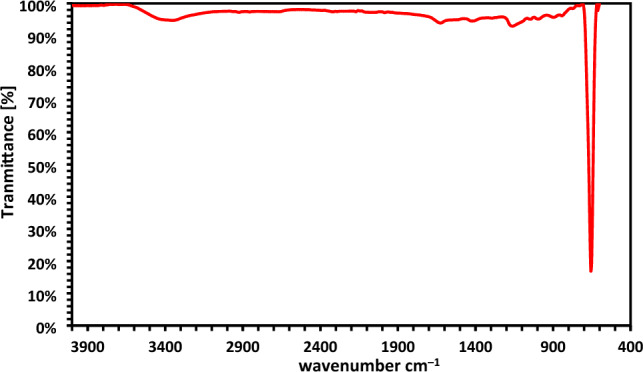
FTIR analysis of Co_3_O_4_NPs.

The purity of the Co_3_O_4_ nanoparticles as they were created, as well as the formation of metal oxide nanoparticles were both confirmed by the FTIR spectrum of a Cobalt oxide nanoparticle, which displayed infrared absorption peaks revealed the vibrational modes at 655 cm^–1^ (Fig. 1). The band that occurred at 655 cm^–1^, specifically, can be attributed to the M–O bond's stretching vibration mode in which M, Co^2+^, is tetrahedrally coordinated^35–37^. The C–O–C symmetric stretch vibration may be indexed to the band at 1180.3 cm^–1^. The peak that developed between 3300 and 3600 cm^–1^ is attributed to O–H stretching, whereas the minor absorption peak at 1635.45 cm^–1^ is connected to the bending vibration of the adsorbed water and surface hydroxyl group.

#### X-ray diffraction (XRD)

**Figure 2:**
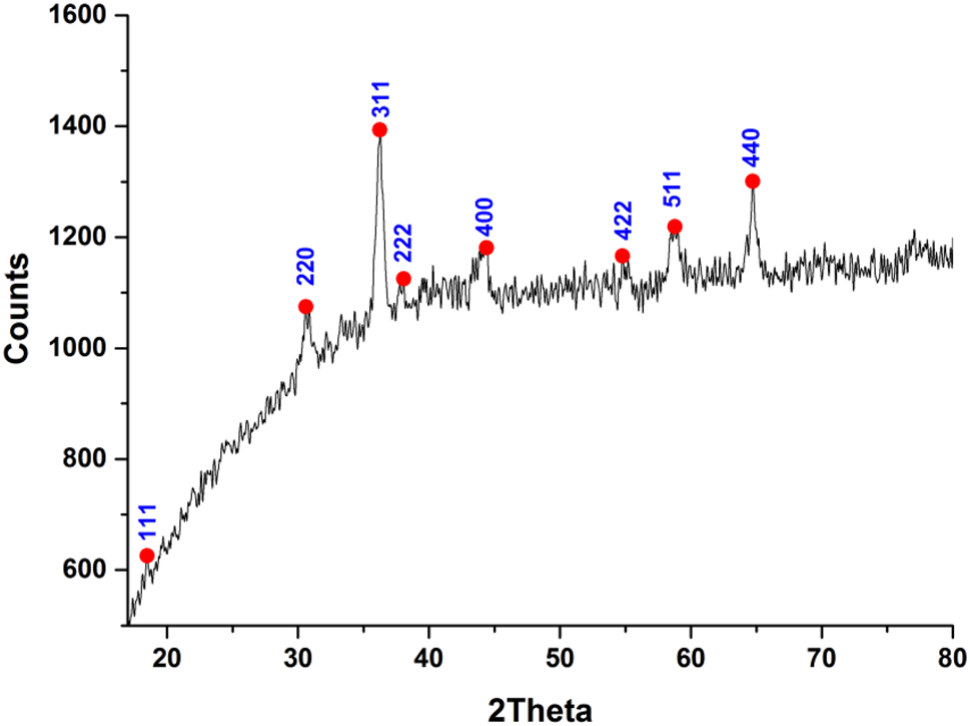
XRD of Co_3_O_4_NPs.

According to the acquired spectrum, the peaks in the XRD spectrum are associated with the face-centered cubic (FCC) structures and correspond to the JCPDS card No. 071-1178 for cobalt oxide (Fig. 2). The prominent peaks were found at 19, 30.6°, 36.29°, 39°, 43.81°, 56.4°, 58.5° and 64.69°, which show the lattice scattering plane. The planes of (111), (220), (311), (222), (400), (422), (511) and (440) can be indexed to a spinel Co_3_O_4_ cubic structure (JCPDS No. 43-1003)^36,37^. No cobalt metal peak can be detected in this pattern, indicating that the Co_3_O_4_ is 100 percent pure. These diffraction bands are generally sharp, symmetrical, and narrow with a low and stable baseline, indicating that the Co_3_O_4_NPs is single phased in the cubic crystal structure and confirming the high purity of the existing material, which may be attributed to the calcination impact and that the cobalt salt was completely converted into Co_3_O_4_NPs. These results were reliable with the values stated in the literature^38,39^.

#### X-ray photoelectron spectroscopy (XPS)

**Figure 3:**
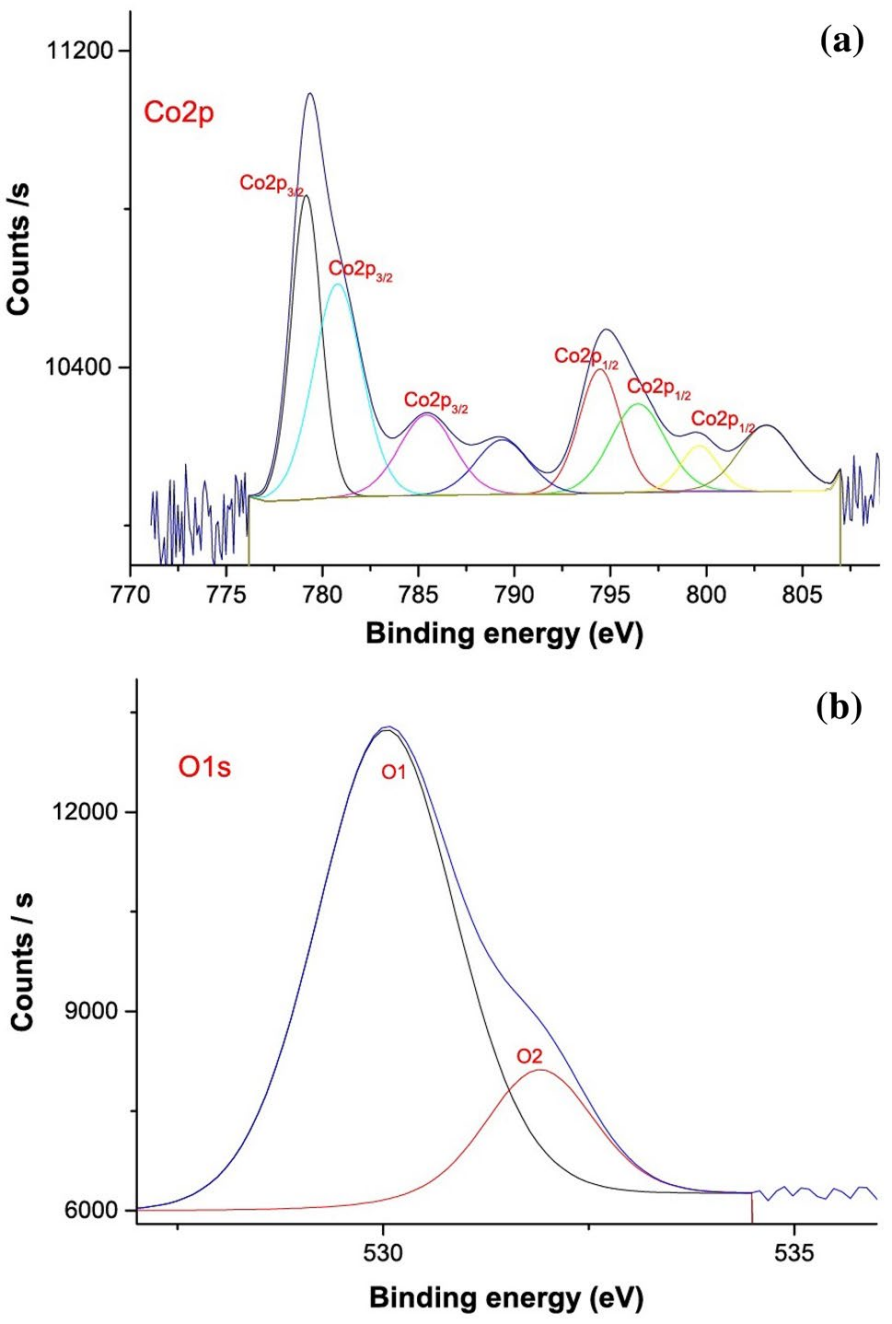
XPS analysis of (**a**) Co 2p, and (**b**) O1s of the Co_3_O_4_ Nps.

X-ray photoelectron spectroscopy (XPS) was used to do more research on these materials' chemical makeup^40–47^. A low and a high energy band, corresponding to Co 2p_3/2_ and Co 2p_1/2_, with two satellites, are visible in Fig. 3a's Co 2p spectrum. These bands are indicative of Co_3_O_4_ phases since they are located at 779.2 and 796.1 eV. The Co 2p_1/2_ peak of 794.2 eV may be further deconstructed into two fitting peaks at 796.1 and 798.6 eV, while the Co p_3/2_ peak of 779.2 eV can be further disassembled into two fitting peaks at 780.8 and 785.4 eV. Co^3+^ may be assigned to the peaks with the binding energies of 779.2 and 794.2 eV, whereas Co^2+^ can be assigned to the peaks with 780.8 and 796.1 eV. The presence of lattice oxygen and surface oxygen on the surface is further confirmed by the high-resolution O 1s spectra in Fig. 3b, which can be further decomposed into two fitting peaks at 530.3 and 531.9 eV. By promoting the creation of oxygen vacancies (Ov) that contained localised electrons, it was thought that Os contributed to the generation of reactive oxygen species (ROS).

#### Thermal gravimetric analysis (TGA)

**Figure 4:**
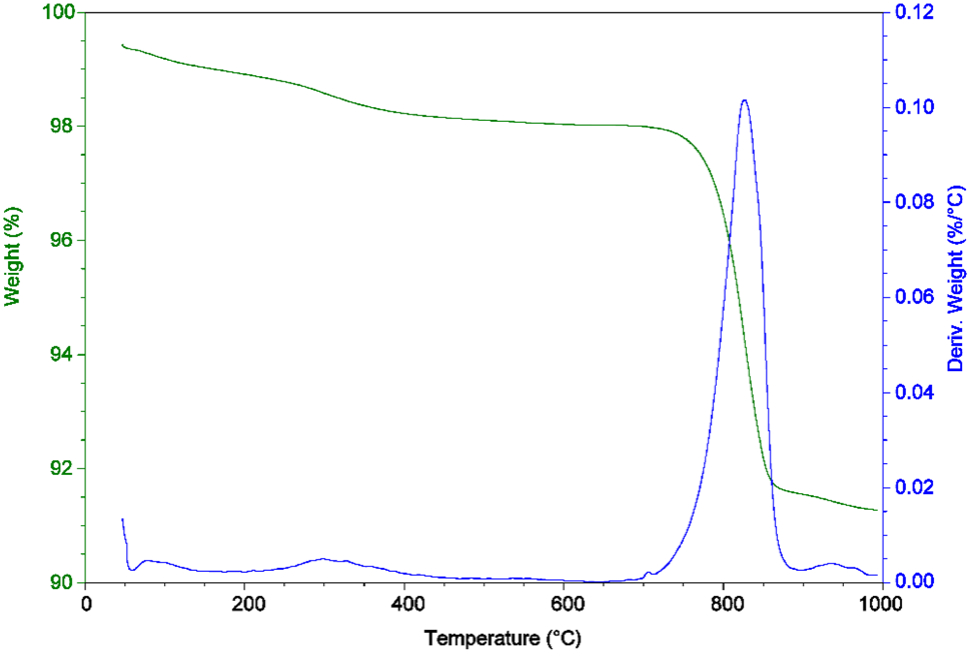
TGA and DTA analyses of Co_3_O_4_NPs.

Co_3_O_4_NPs are degraded in four phases and are subjected to TGA and DTA studies in Fig. 4. In the temperature range of 25–180 °C, the first step is ascribed to the removal of adsorbed/trapped water, and the mass loss for this phase is determined to be 0.515%^35,45–47^. Within the temperature range of 180–450 °C, the second stage results in a loss of 0.761%. The third stage is carried out in the 450–900 °C temperature range, which is responsible for the maximum mass loss of 6.594%. The fourth step of weight loss occurs in the temperature range of 900–1000 °C, which is attributed to the lowest mass loss of 0.282%. Co_3_O_4_NPs (91.848%) constitute the final mass loss's remnant. The results of the TGA curves were corroborated by DTA curves, which showed three endothermic bands at 79.00, 298.81, 826.68, and 934.94 °C.

#### *Scanning electron microscopy (SEM)* and *transmission electron microscopy (TEM)*

**Figure 5:**
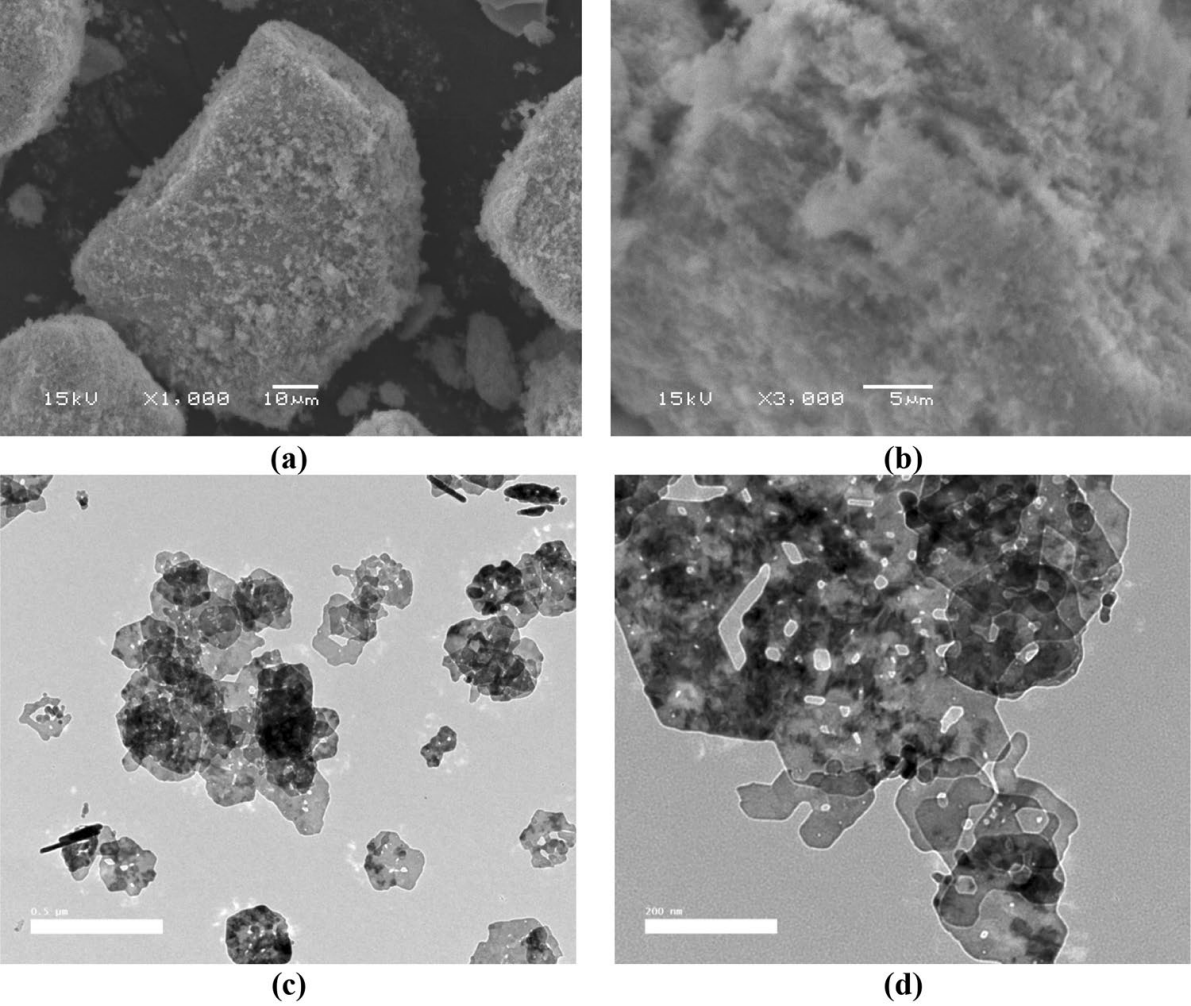
(**a**,**b**) SEM, (**c**,**d**) TEM of Co_3_O_4_NPs images.

Figure 5a,b displays typical SEM images of synthetic Co_3_O_4_ particles. The development of the samples' morphology was looked at. The substance had a sphere-like shape, and assembled spheres agglomerated together, representing consistent homogeneity and a strong connection between the grains. The shape of the Co_3_O_4_ nanoparticles was homogenous, and the SEM pictures showed that the Co_3_O_4_ nanoparticles were created throughout the synthesis process by aggregating smaller crystallites^35,36^.

The TEM picture of the Co_3_O_4_ nanoparticles is shown in Fig. 5c,d. Most particles have a spherical form and a restricted size distribution. The majority of spherical particles are found on surfaces that are organised and aggregated in cubic shapes.

#### *Surface area and porosity of Co*_*3*_*O*_*4*_*NPs*

Table 3 shows the Co_3_O_4_NPs porosity and Surface area (SA). The surface area analysis was carried out on Co_3_O_4_ nanoparticles by the BET method. The BET surface area of Co_3_O_4_NPs, estimated by the multipoint BET model, is 30.312 m^2^/g, 16.55 nm mean pore diameter (MPD), and 0.1375 cm^3^/g total pore volume (TPV), assuming the particles have solid, varied shapes and sizes.

**Table 3:** Co_3_O_4_NPs' porosity and surface area were obtained by BET analysis.

Sample	BET SA (m^2^/g)	MPD (nm)	TPV (cm^3^/g)
Co_3_O_4_NPs	30.312	16.55	0.1375

### The impact of Co_3_O_4_NPs on biogas and biomethane production

**Figure 6:**
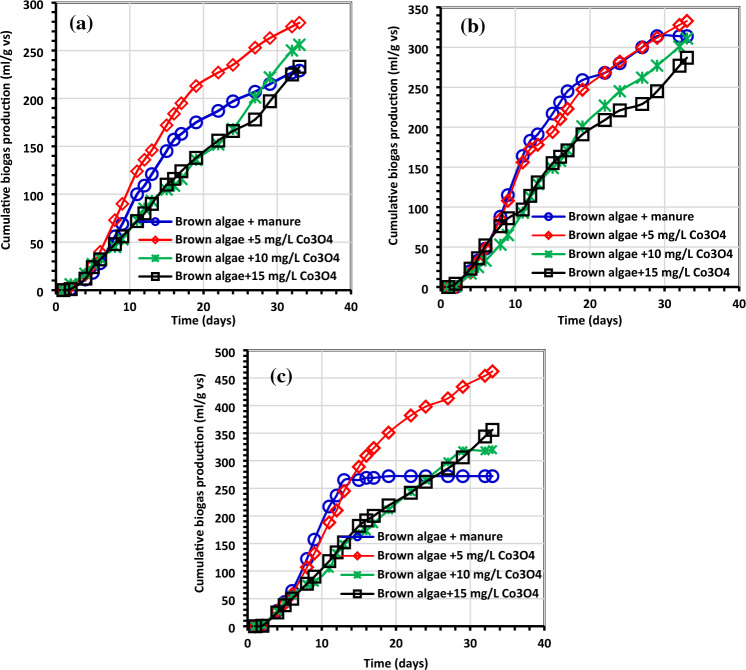
Average cumulative net biogas generation (mL/g VS) using (**a**) *C. myrica* (0.5 gm) treated with various amounts of Co_3_O_4_NPs, (**b**) *C. myrica* (1.0 g) treated with various amounts of Co_3_O_4_NPs and (**c**) *C. myrica* (1.5 g) treated with various amounts of Co_3_O_4_NPs.

**Figure 7:**
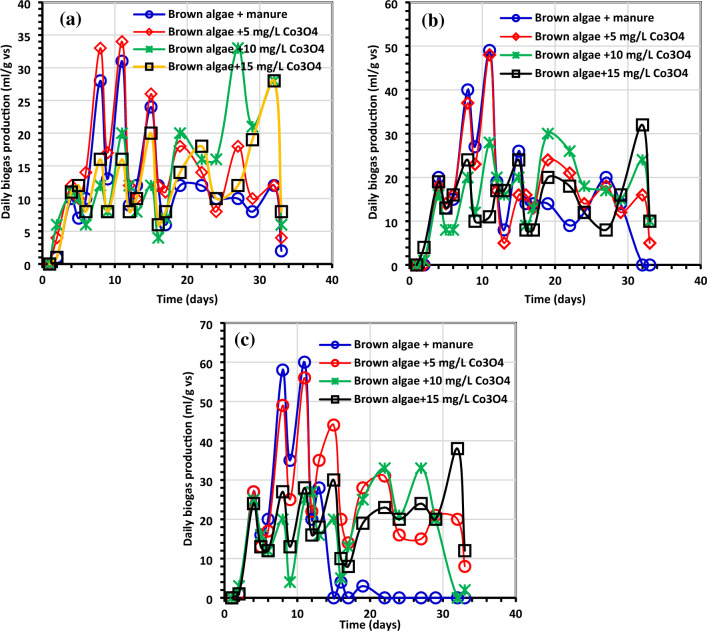
Average daily biogas generation (mL/g VS) using (**a**) *C. myrica* (0.5 g) treated with various amounts of Co_3_O_4_NPs, (**b**) *C. myrica* (1.0 g) treated with various amounts of Co_3_O_4_NPs and (**c**) *C. myrica* (1.5 g) treated with various amounts of Co_3_O_4_NPs.

**Figure 8:**
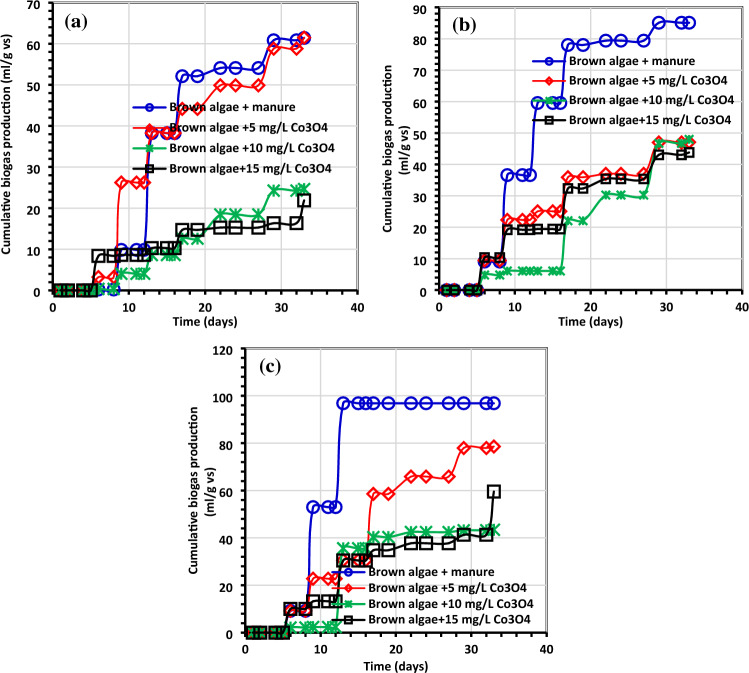
Average net cumulative methane generation (mL/g VS) using (**a**) *C. myrica* (0.5 g) treated with various amounts of Co_3_O_4_NPs, (**b**) *C. myrica* (1.0 g) treated with various amounts of Co_3_O_4_NPs and (**c**) *C. myrica* (1.5 g) treated with various amounts of Co_3_O_4_NPs.

**Figure 9:**
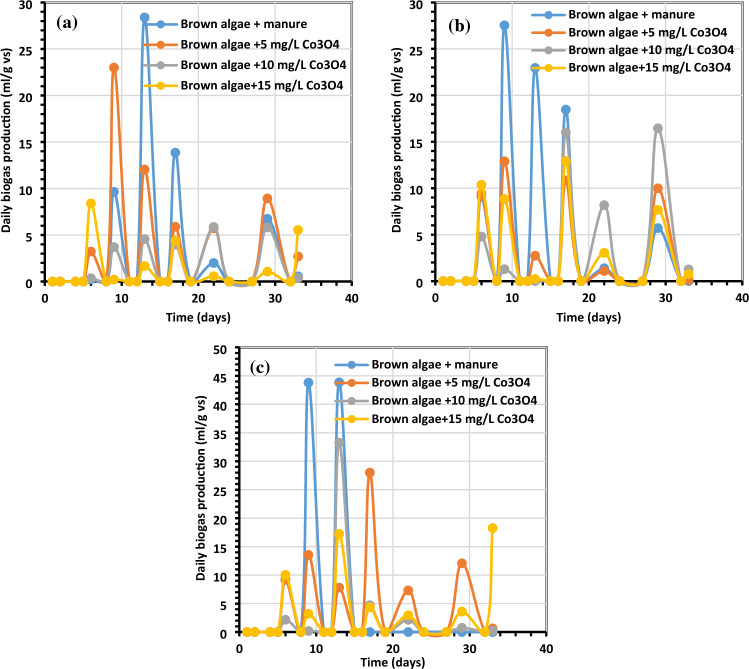
Methane output average daily (mL/g VS) using (**a**) *C. myrica* (0.5 g) treated with various amounts of Co_3_O_4_NPs, (**b**) *C. myrica* (1.0 g) treated with various amounts of Co_3_O_4_NPs and (**c**) *C. myrica* (1.5 g) treated with various amounts of Co_3_O_4_NPs.

Figures 6, 7, 8 and 9 display the experimental findings of biogas and biomethane yield, which were gathered during 33 days. The lowest Co_3_O_4_NPs doses, 5 mg/L, were optimum for all treatments. As shown in Fig. 6, the average biogas production yield for *C. myrica* treated with Co_3_O_4_NPs dose (5 mg/L) was slightly higher with values of 279, 333, and 462 mL/g VS for 0.5, 1 and 1.5 g of *C. myrica* when compared to 229, 314 and 272 mL/g VS for the untreated *C. myrica*.

The maximum biogas was produced when 1.5 g of *C. myrica* was treated with 5 mg/L of Co_3_O_4_NPs, and this ought to be mentioned. The use of 5 mg/L of Co_3_O_4_NPs on *C. myrica* had a significant favorable impact on the generation of biogas (p < 0.05). Moreover, when *C. myrica* was treated with Co_3_O_4_NPs (10 and 15 mg/L), higher biogas yield was achieved, more than the control for all treatments except for 15 mg/L of Co_3_O_4_NPs with 1.0 g of *C. myrica* when 287 mL/g VS biogas was acquired, which is slightly lower than control when 314 mL/g VS of biogas was achieved. From the above data, it can be noticed that the biogas production from *C. myrica* followed a specific trend in all experiments where the positive effect of Co_3_O_4_NPs on biogas production, especially for lower concentrations, but this enhancement may increase with the increasing of the biomass amount.

On the other hand, unlikely the biogas production, the biomethane yield is prohibited by the addition of Co_3_O_4_NPs except for the lower Co_3_O_4_NPs dosages (5 mg/L) with low *C. myrica* 0.5 g, which has biomethane yield of 61.48 mL/g VS near the control yield 61.40 mL/g VS (Fig. 8). For the rest of the treatment by increasing the Co_3_O_4_NPs dosages (10 and 15 mg/L), a decrease in biomethane can be achieved to reach less than 33%.

Our findings are consistent with those of Ali et al.^25^, who looked at how (Co_3_O_4_NPs) supplementation affected anaerobic microbial population changes, anaerobic digestion (AD) performance, and anaerobic digestion (AD) output. Co_3_O_4_NPs (3 mg/L) demonstrated the most significant improvement in biogas output over the cow dung as control and the co-digestion process of manure with water hyacinth by 58.9 and 27.2%, respectively. Additionally, increasing the dosage of Co_3_O_4_ up to 5 mg/L resulted in a drop in biogas and biomethane compared to the control^25^. In this study, the biogas increased in all treatments with Co_3_O_4_ (5 mg/L) while the CH_4_ decreased for 5, 10 and 15 mg/L, which also agrees with Ali et al.^25^, who started only with 1, 3 and 5 mg/L.

The greatest biogas output in our investigation, at 462 mL/g VS, was somewhat lower than that reported by Hassaan et al.^4^, who investigated the impact of ozonation pretreatment on the generation of biogas from *U. lactuca*, with a biogas yield of roughly 499 mL/g VS and a 30-min ozonation duration. This research can be a worthy addition to confirm that not all biogas can be economical, and the type of each nanoparticle needs further investigations to declare which stage of AD can have its impact. In this study, it can be noticed that biogas increased from 320 mL/g VS for Co_3_O_4_NPs dosage 10 mg/L to 356 mL/g VS for Co_3_O_4_NPs dosages 15 mg/L starting from day 24 for 1 g of *C. myrica*. For most of Co_3_O_4_NPs dosages treatment, the increase of biogas yield started from day 13, while the highest biogas yield for all control treatments was during the first 10 days. Moreover, for methane production from the control samples, the lower amounts of *C. myrica* 0.5 and 1.0 g have an increase in methane production with 3 dominant peaks during the first 20 days. But for a higher amount of *C. myrica* (1.5 g) these peaks decrease to reach their maximum in the first 15 days and the third peak is substituted by the lower dosage of Co_3_O_4_NPs (5 mg/L). It can be noticed that the increase in the amount of biomass of 1.0 g has an optimistic influence on biogas generation, which is nearly 35.5% of the total biogas 272 mL/g VS.

The results demonstrated that the system of biogas and biomethane production needs more optimization and adjustment, especially the nanoparticles amount and biomass amount that may lead to enhancing the biomethane production from the final biogas. Further investigation to study the genes of manure bacteria is still needed to determine which one is responsible for methanogenesis.

To investigate the impacts of Co NPs (1.4, 2.7, and 5.4 mg/L) added during AD of poultry litter, Hassanein et al.^48^ carried out an experiment. Compared to the control, methane generation increased by adding NPs. The reactors treated with 5.4 mg/L of Co showed the most significant increases, which were 29.7% in each case. On the other hand, Co_3_O_4_ NPs were shown to have favourable effects at lower concentrations (1.0 mg/L), and this might be because the trials were conducted under different settings^49^. In our work (5, 10, 15 mg/L) the lower concentrations of Co_3_O_4_ (5 mg/L) are favourable in biogas production while biomethane in the control gave the highest yield of 96.85% mL/g VS. Our work also agrees with Gran et al.^49^ where low concentrations of Co_3_O_4_ gave the highest biogas production. The different forms of the Co_3_O_4_NPs, biomass and types of manure may be the reasons for such differences.

Using NPs positively benefits effluent quality, process optimisation, and CH_4_ generation. When present at an ideal concentration (e.g., 100 mg/L of Fe NPs, 2 mg/L of Ni NPs, and 1 mg/L of Co NPs), their role as essential nutrient suppliers helps to facilitate the manufacture of essential enzymes and co-enzymes, which in turn stimulates anaerobic microorganism activities^50^. Additionally, the generation of CH_4_ is decreased when using Co NPs at concentrations greater than 2 mg/L and 1 mg/L, respectively. Combining Fe/Ni/Co NPs is more effective than single NPs at increasing CH_4_ generation. In order to increase the efficiency of anaerobic digestion, NPs combinations can be used as supplements.

Glass and Orphan^51^ found that cobalt in cobamides (Cobamide is a naturally occurring chemical compound containing cobalt in the corrinoid family of macrocyclic complexes) is responsible for transferring the methyl group throughout all methanogenesis routes (methylotrophic, acetotrophic pathways, and hydrogenotrophic), and that Co is a necessary metal to boost metalloenzyme activity during each of these processes. Low Co_3_O_4_ concentrations were found in this investigation, which is consistent with^14^, who claimed that reduced Co-NPs (1 mg/L) improved biogas and methane output during 50 days. Additionally, they discovered that adding Co-NPs to the diet reduced both the daily maximum production time and the bacterial lag phase, consistent with this study's findings that all lag phases were reduced compared to controls (Tables 4, 5).

**Table 4:** Kinetic investigation of biogas using the modified Gompertz model.

	*R*^2^	Expected *P* (mL/g VS)	Variances (%)	*R*_m_ mL/gVS day	λ (day)
0.5 g *C. myrica* + Co_3_O_4_NPs					
Control	0.996	225.78 + 3.18	3.71	10.16 + 0.17	0.16 + 0.001
*C. myrica* + 5 mg/L Co_3_O_4_NPs	0.996	277.48 + 4.29	3.55	10.09 + 0.19	0.15 + 0.007
*C. myrica* + 10 mg/L Co_3_O_4_NPs	0.991	376.54 + 37.54	2.01	19.35 + 1.75	0.07 + 0.01
*C. myrica* + 15 mg/L Co_3_O_4_NPs	0.989	262.63 + 14.98	5.55	14.19 + 0.081	0.09 + 0.01
1.0 g *C. myrica* + Co_3_O_4_NPs					
Control	0.995	311.13 + 4.35	2.14	9.14 + 0.17	0.18 + 0.01
*C. myrica* + 5 mg/L Co_3_O_4_NPs	0.992	331.23 + 7.69	3.91	10.12 + 0.28	0.15 + 0.01
*C. myrica* + 10 mg/L Co_3_O_4_NPs	0.997	334.42 + 7.57	3.73	13.25 + 0.28	0.11 + 0.01
*C. myrica* + 15 mg/L Co_3_O_4_NPs	0.989	295.95 + 11.59	6.24	11.57 + 0.51	0.11 + 0.01
1.5 g *C. myrica* + Co_3_O_4_NPs					
Control	0.994	276.08 + 2.91	1.49	7.05 + 0.13	0.35 + 0.02
*C. myrica* + 5 mg/L Co_3_O_4_NPs	0.998	458.30 + 5.41	3.48	10.38 + 0.14	0.16 + 0.01
*C. myrica* + 10 mg/L Co_3_O_4_NPs	0.995	367.59 + 11.51	1.52	12.82 + 0.41	0.10 + 0.01
*C. myrica* + 15 mg/L Co_3_O_4_NPs	0.990	381.83 + 17.15	5.65	12.86 + 0.60	0.10 + 0.01

**Table 5:** Methane kinetic data were analyzed using a modified Gompertz model.

	*R*^2^	Expected P (ml/g VS)	Variances (%)	*R*_m_ mL/gVS day	λ (day)
Algae 0.5 g + Co_3_O_4_NPs					
Control	0.965	58.69 + 2.20	4.55	12.28 + 0.38	0.32 + 0.06
*C. myrica* + 5 mg/L Co_3_O_4_NPs	0.958	56.80 + 2.56	8.58	10.22 + 0.53	0.20 + 0.03
*C. myrica* + 10 mg/L Co_3_O_4_NPs	0.978	27.91 + 2.17	0.68	16.25 + 0.91	0.12 + 0.02
*C. myrica* + 15 mg/L Co_3_O_4_NPs	0.879	18.37 + 1.70	19.29	9.02 + 1.17	0.13 + 0.03
Algae 1.0 g + Co_3_O_4_NPs					
Control	0.973	84.54 + 2.74	1.10	9.79 + 0.38	0.24 + 0.03
*C. myrica* + 5 mg/L Co_3_O_4_NPs	0.943	46.02 + 2.89	5.53	10.09 + 0.76	0.15 + 0.03
*C. myrica* + 10 mg/L Co_3_O_4_NPs	0.947	71.77 + 19.42	1.35	22.06 + 3.76	0.09 + 0.03
*C. myrica* + 15 mg/L Co_3_O_4_NPs	0.939	45.75 + 3.86	2.86	11.32 + 1.05	0.12 + 0.02
Algae 1.5 g + Co_3_O_4_NPs					
Control	0.957	98.61 + 3.29	1.81	9.09 + 0.37	0.40 + 0.08
*C. myrica* + 5 mg/L Co_3_O_4_NPs	0.961	84.07 + 5.89	0.11	13.21 + 0.80	0.14 + 0.02
*C. myrica* + 10 mg/L Co_3_O_4_NPs	0.985	41.91 + 0.77	5.81	12.35 + 0.10	3.01 + 0.62
*C. myrica* + 15 mg/L Co_3_O_4_NPs	0.913	47.31 + 3.84	23.18	11.02 + 0.95	0.15 + 0.04

From the above-mentioned result, it is essential to conclude that the impact of CoNPs mainly depends on the following: (1) the type and the nature of the CoNPs where its Co only, or CoO NPs, Co_3_O_4_ and maybe also depend on its size and surface area and the way of synthesis because the only study that published Co_3_O_4_^25^ did not synthesize it and purchased the material where it has SA of 46.5 m^2^/g, which is greater than synthesized Co_3_O_4_ in this study which has SA of 30.312 m^2^/g. (2) the dosage optimization of the nanoparticles will depend on the nature of the element, especially when using the trace elements such as Co and Ni, which are very critical in enzymatic activities and are different from Fe where its concentrations can reach more than 100 mg/L and did not show any side effects on the AD process. (3) Co_3_O_4_ nanoparticles may form aggregates in the biodigester, which can hinder their interaction with the microbial community responsible for methane production. Aggregation can limit the number of catalytic sites available for the reaction, thereby reducing the process's overall efficiency. (4) The substrate type and the amount that may need to be optimized depending on C/N ratio and other precautions depending on the type where algal biomass is different from agricultural biomass. (5) Finally, the composition of biogas and its contents will vary depending on the substrate type and the NPs dosage and not all biogas will have a high content of CH_4_ and may produce ammonia or H_2_S depending on the substrate type, and the impact of NPs may affect the special type of bacterial or prevent the conversion of CO_2_ to CH_4_ in one or more of hydrogenotrophic, methylotrophic, and acetotrophic pathways. (6) The long-term impacts of Co_3_O_4_NPs on the microbial communities involved in methane production remain unknown. Prolonged exposure to nanoparticles may alter the microbial diversity and composition, affecting the biogeochemical cycles in the environment. (7) Cobalt nanoparticles are known to have potential toxicity to humans and the environment. In specific concentrations, they may pose a risk to the health of microbial communities, leading to reduced methane production.

### Proposed mechanism of Co_3_O_4_ impact on direct electron transfer during biomethane production

**Figure 10:**
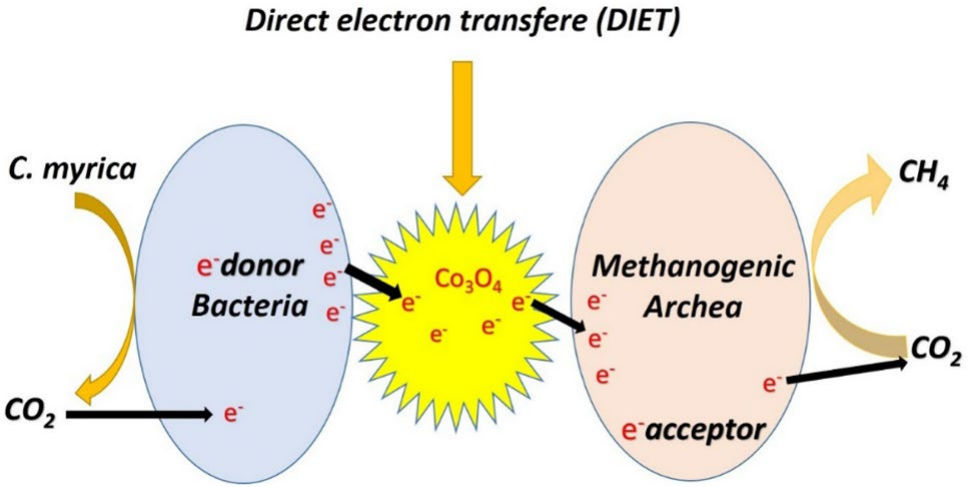
The proposed role of Co_3_O_4_ in electron transfer mechanism.

The following stages may be used to illustrate how this study's findings regarding the suppression of CH_4_ by increasing the dose of Co_3_O_4_ NPs:

I. When added to the AD process, higher Co_3_O_4_ NPs dosages may have an inhibiting influence on the formation of biogas. Cobalt is needed by metalloenzymes in bacteria that generate CH_4_, claim Ali et al.^25^. Through the creation of enzymes and metabolic processes, trace metals including Co, Ni, and Fe are necessary for the development and activity of methanogenic bacteria. The production and stability of biogas are also significantly impacted by certain macronutrients and trace elements^14,50^. Because trace metals are needed by cofactors and enzymes, it has been demonstrated that supplementing with them during AD can stabilize and enhance digestive capacity. According to Luna-delRisco et al.^16^, the fundamental metabolic mechanisms of the AD process depend on the incidence of heavy metal ions through organic matter (OM). High concentrations of these micronutrients, however, may prevent proper biodegradation. Glass and Orphan^51^ found that cobalt in cobamides is responsible for the transfer of the methyl group throughout all methanogenesis routes (hydrogenotrophic, methylotrophic, and acetotrophic pathways) and that Co is a necessary metal to boost metalloenzyme activity during each of these processes.
II. The higher concentration of Co_3_O_4_ nanoparticles may agglomerate and obstruct their contact with the microbial population that produces methane. Aggregation can lower the total efficiency of the process by limiting the number of catalytic sites accessible for the reaction.
III. The organic matter (substrate) conversion by microorganisms into intermediate products, including acetate, hydrogen, and carbon dioxide through AD is the electron transfer process of methane synthesis in the presence of cobalt in biogas. Enzymes involved in converting organic materials to methane work with cobalt as a co-factor. Volatile fatty acids (VFAs), H_2_, and CO are reduced molecules that are produced during the oxidation of organic materials (Fig. 10). As a result of the subsequent oxidation of these reduced molecules, CO_2_, H_2_O, and other reduced molecules such hydrogen sulfide (H_2_S) and methane (CH_4_) are created. The reduction of H_2_ and CO_2_ to generate methane is the result of the electron transfer process, which includes the transfer of electrons from reduced molecules to electron acceptors like these. By providing a necessary ingredient for coenzyme B12, which is engaged in the last stage of the methane synthesis process, cobalt aids in the creation of methane. In general, the electron transfer process of methane generation in the presence of cobalt in biogas entails the organic matter conversion to methane by the oxidation and reduction of molecules by microbes with the assistance of cobalt as a co-factor for coenzyme B12.
IV. Eqs. (2–4) may be used to depict the electron transfer equation for generating methane from organic matter in the presence of Co_3_O_4_ (Fig. 10).
  2 $$ {\text{Co}}_{{3}} {\text{O}}_{{4}} + {\text{ 4H}}_{{2}} {\text{O }} + {\text{ 8e}} \to {\text{4OH}}^{-} + {\text{ CO}}_{{2}} $$
  3 $$ {\text{Organic matter }} + {\text{ 4H}}_{{2}} {\text{O }} + {\text{ 8OH}}^{-} \to {\text{CH}}_{{4}} + {\text{ 8OH}}^{-} $$
  4 $$ {\text{Overall equation}}:{\text{Co}}_{{3}} {\text{O}}_{{4}} + {\text{ Organic matter}} \to {\text{CH}}_{{4}} + {\text{ CO}}_{{2}} $$

Co_3_O_4_ serves as an electron acceptor in this process, transferring electrons from the organic material to water molecules to create hydroxide ions. In the meantime, CH_4_ and CO_2_ are produced during the oxidation of the organic materials.

V. By giving microorganisms a surface to cling to, Co_3_O_4_NPs can improve microbial activity and speed up the transfer of electrons.
VI. Higher Co_3_O_4_ nanoparticle concentrations may harm the electron transfer processes necessary to produce methane from organic materials. This is due to the ability of Co_3_O_4_ nanoparticles to serve as electron traps, which obstruct the transport of electrons to the target molecules and hence impede the process as a whole. The nanoparticles can physically block the target molecules' ability to diffuse, hindering the electron transfer process even more. Co_3_O_4_ nanoparticles' detrimental effects on electron transfer processes might ultimately reduce the yield and efficiency of methane generation.
VII. In particular, Co_3_O_4_ nanoparticles may interfere with the activities of the bacteria responsible for the creation of methane. The conversion of carbon dioxide and other chemicals into methane by methane-producing bacteria depends on electron transfer processes. The efficiency of methane synthesis may be decreased or stopped entirely if Co_3_O_4_ particles obstruct these processes. Furthermore, Co_3_O_4_ nanoparticles may poison the bacteria responsible for creating methane. This may lead to a decline in the microbial population's general activity, which might harm methane generation. Overall, it is evident that Co_3_O_4_ nanoparticles may interfere with electron transfer processes and decrease the efficiency of this process, even if further research is required to thoroughly understand the possible adverse effects of these particles on methane synthesis.

### The proposed role of Co_3_O_4_ in the formation of reactive oxygen species (ROS) and its impact on the reduction of biogas and methane

Increasing the system's Co_3_O_4_NPs content could increase electron transfer efficiency, raising the pace at which biogas is produced and lowering methane emissions. This is because Co_3_O_4_NPs can hasten the organic matter conversion to biogas by improving the kinetics of electron transfer processes. It's crucial to remember, too, that adding too many Co_3_O_4_NPs might also have a detrimental impact on the system's performance. For instance, a barrier that stops microorganisms from accessing the electrode surface may be created if the Co_3_O_4_NPs concentration rises too high, resulting in slower electron transfer rates^25,49,50^.

Additionally, too much Co3O4 might produce ROS, which can injure microorganisms and decrease their activity^26,52,53^. As a result, the system's efficiency may suffer, and biogas production rates may decline. Co_3_O_4_-based biogas generation involves a complicated process that can result in ROS development through various reactions^50^. The Eqs. (5–7) that explain the potential routes are listed below.

5 $$ {\text{Co}}_{{3}} {\text{O}}_{{4}} + {\text{ H}}_{{2}} {\text{O }} \to {\text{ CoO }} + {\text{ 2 H}}^{ + } + { 1}/{\text{2 O}}_{{2}} + {}^{ \cdot }{\text{OH}} $$

This equation describes the reaction between Co_3_O_4_ and water, which can generate hydroxyl radicals (^•^OH), a type of ROS.

6 $$ {\text{Co}}_{{3}} {\text{O}}_{{4}} + {\text{ O}}_{{2}} \to {\text{ 3 CoO }} + { 1}/{\text{2 O}}_{{2}}^{{ \cdot {-}}} + {\text{ H}}_{{2}} {\text{O}}_{{2}} $$

The formation of superoxide radicals (O2^•–^) and H_2_O_2_, both of which constitute ROS, is described by this equation. This reaction can be catalysed by Co_3_O_4_, resulting in the production of ROS.

7 $$ {\text{Co}}_{{3}} {\text{O}}_{{4}} + {\text{ 2 H}}^{ + } + {\text{ 2 e}}^{-} \, \to {\text{ 3 CoO }} + {\text{ H}}_{{2}} {\text{O}} $$

By transferring electrons, Co_3_O_4_NPs is reduced to CoO according to this equation. H + protons and H2O can both be produced by this reaction. Although this reaction doesn't directly create ROS, it can cause the formation of ROS in other CoO or Co_3_O_4_NPs-based reactions. The ROS generated during the biogas generation process might have both beneficial and bad effects. ROS may function in the biogas generation process as signalling molecules that control microbial activity^53^. Increased generation of biogas can result from ROS's ability to trigger the gene expression related to metabolism and the stress response. Additionally, ROS can interact with extracellular polymeric substances (EPS) produced by microbes, which can strengthen cell adhesion to electrode surfaces and boost electron transfer rates.

On the other hand, excessive ROS production can harm microorganisms and cause oxidative stress, which can lower their activity and impair the production of biogas^26^. This is due to the fact that ROS may interact with molecules found in cells, including lipids, proteins, and DNA, causing denaturation of proteins, DNA mutations, and damage to cell membranes. Cell death and decreased generation of biogas may result in the end result of this. The formation of ROS throughout the biogas production process must thus be properly balanced. Co_3_O_4_NPs is a catalyst that may be used to produce ROS, as well as other processes, including exposure to light or extreme temperatures. It is feasible to optimize ROS formation to increase biogas generation while minimizing the adverse consequences of oxidative stress by meticulously managing system parameters like the pH and catalyst concentration. In rare circumstances, ROS may contribute to a decrease in the generation of methane and biogas. This happens when there is excessive ROS generation, which causes oxidative stress and harm to the microorganisms used in biogas production.

The primary component of biogas, methane, is created when microbes break down organic material in anaerobic conditions. Changes in the environment, especially the presence of ROS, can have an impact on the methane generation process. ROS may interact with lipids, proteins, and DNA in bacteria to cause cell death and damage^26^. This may eventually cause the microorganisms involved in the creation of biogas to become less active, which would reduce the generation of both biogas and methane. The oxidation of methane and the creation of other substances like CO_2_ and water can also be caused by the reaction of ROS with methane. Methane may naturally undergo this oxidation process in some conditions, such as the atmosphere, where it combines with hydroxyl radicals to produce water and carbon dioxide.

As a result, while ROS can encourage microbial activity and electron transfer, which can help with biogas production^51^, too much ROS production can also reduce the generation of methane and biogas^26^. To reduce oxidative stress and maximize the generation of biogas and methane, the conditions of the biogas production process must be carefully controlled. It is significant to highlight that additional reactions besides those involving Co_3_O_4_ can also result in the generation of ROS in the production of biogas, and the processes behind these reactions are not fully understood. In order to completely comprehend the procedure and optimize the circumstances for effective biogas generation while minimizing ROS creation, more study is thus required. To ensure that the advantages of the catalyst are maximized while minimizing any potential drawbacks, it is crucial to optimize the amount of Co_3_O_4_ employed in the system. Extensive experimentation and system monitoring are needed to identify the ideal circumstances for Co_3_O_4_ utilization in biogas generation.

### Kinetic study

**Figure 11:**
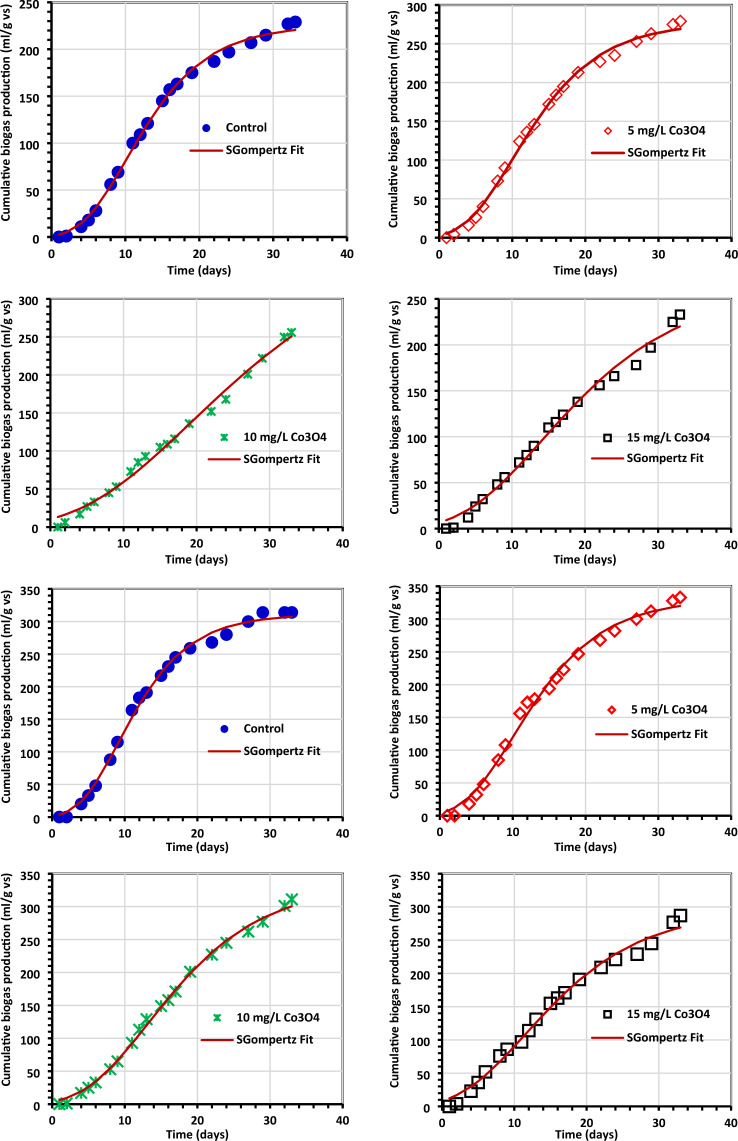

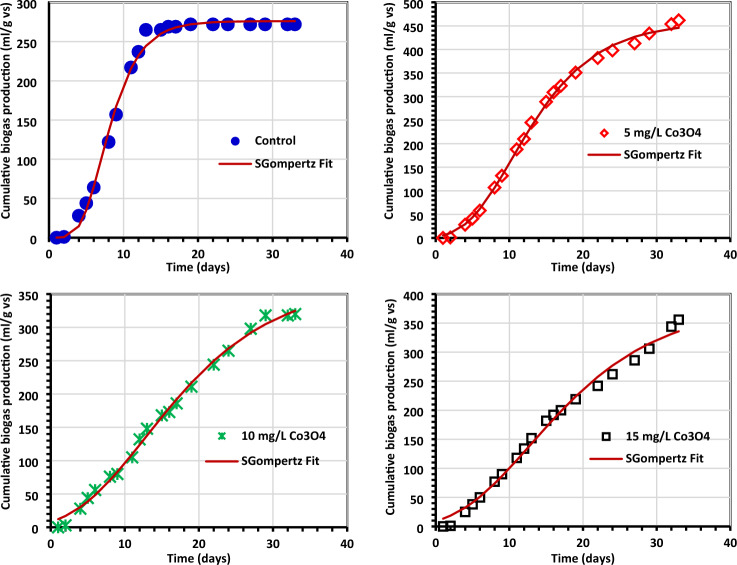
The total biogas yield as determined by the Gompertz model. (**a**) Control (0.5 g of *C.myrica*, (**b**–**d**) control + Co_3_O_4_NPs, (**e**) control (1.0 g of *C. myrica*), (**f**–**h**) Control + Co_3_O_4_NPs, (**i**) control (1.5 g of *C. myrica*) and (**j**–**l**) control + Co_3_O_4_NPs.

**Figure 12:**
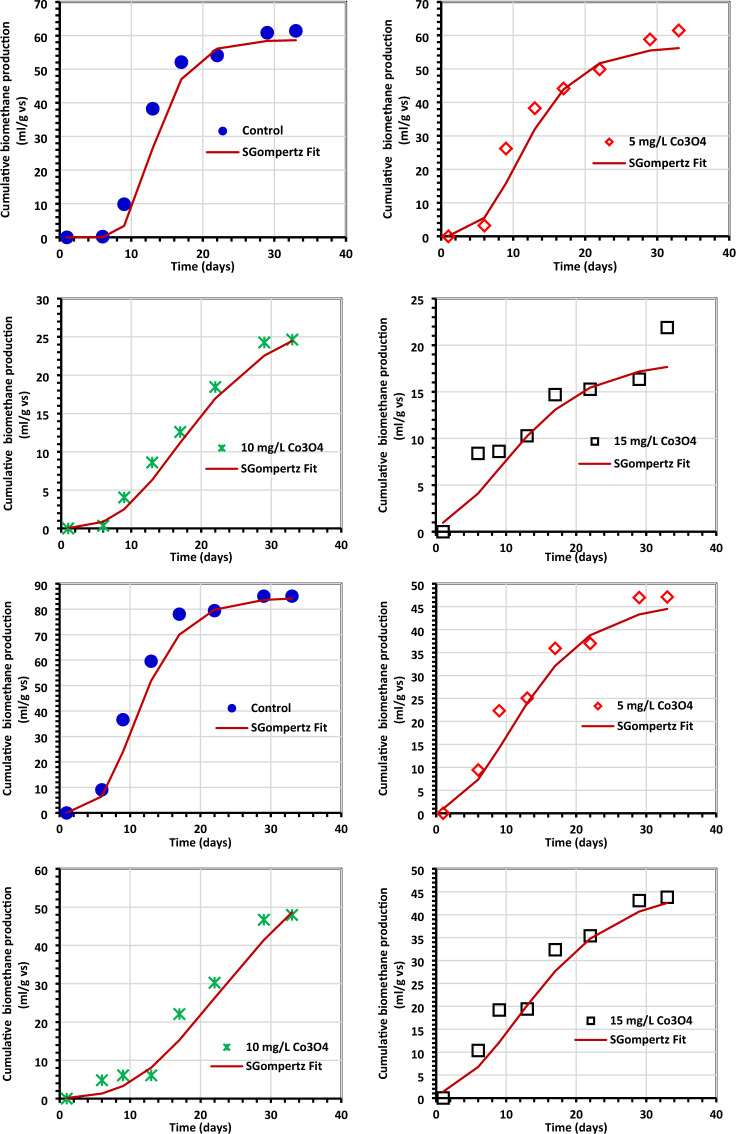

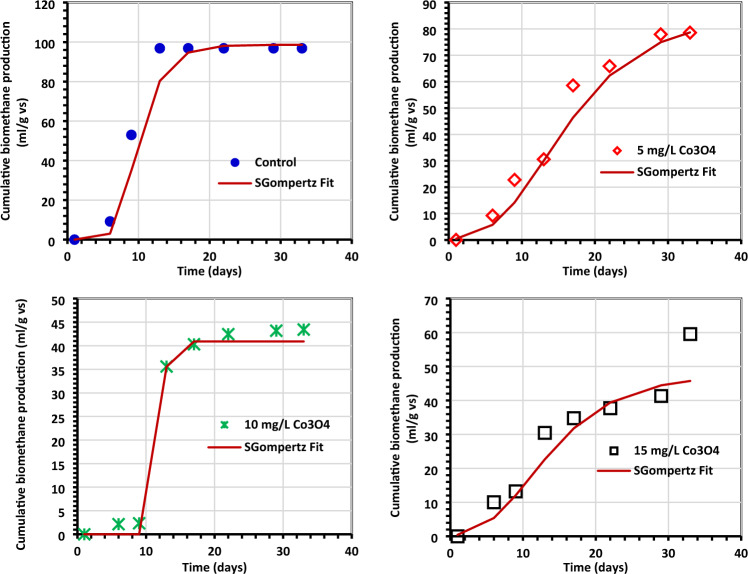
The total methane yield as determined by the Gompertz model (**a**) control (0.5 g of *C. myrica*, (**b**–**d**) control + Co_3_O_4_NPs, (**e**) control (1.0 g of *C. myrica*), (**f**–**h**) Control + Co_3_O_4_NPs, (**i**) control (1.5 g of *C. myrica*) and (**j**–**l**) control + Co_3_O_4_NPs.

Tables 4 and 5 provide an overview of the kinetic data for generating biogas and biomethane, which are displayed in Figs. 11 and 12. The Gompertz model and the experimental findings appeared to match rather well, according to reports. When algae (0.5 g) were treated with 5 mg/L Co3O4NPs, *R*_m_ of 16.35 1.75 mL/g VS of biogas was produced, and when algae (1.0 g) were treated with 10 mg/L Co_3_O_4_NPs, *R*_m_ of 22.06 3.76 mL/g VS of biomethane was produced^33,54^. The modified Gompertz received values of 0.07 ± 0.01 day and 0.09 ± 0.03 for biogas and biomethane production, respectively. This work's value is incredibly low compared to prior values reported for the modified Gompertz model^7,55^ and is the same as those obtained by El Nemr et al.^1^. The reliability of the model was evaluated by comparing the estimated biogas production statistics to the actual values (Fig. 11). To help with the visualization of the kinetics inquiry, additional statistical indicators (*R*^2^) are provided in Table 5. Nguyen et al.^33^ concluded that a better kinetic model was indicated by modified Gompertz models with higher *R*^2^ values (0.999). In our investigation, the Gompertz model has a superior *R*^2^ of 0.998 and 0.985 for the generation of biogas and biomethane, respectively.

### Optimization study

The interaction impacts of three significant parameters, including the amount of biomass, the dosage of Co_3_O_4_NPs, and the contact time on the biogas and biomethane yield were examined using the design matrix. Table 6 displays the experimental setup and the results. The following equations for the generation of biogas and biomethane were established based on the results:

8 $$ \begin{gathered} {\text{Biogas production for Coded Factors }} = {227}.00 + {42}.{\text{13A}}{-}{ 4}0.{\text{87B}} \hfill \\ + { 97}.00{\text{C}}{-}{ 17}.{\text{25AB}} + { 8}.00{\text{AC }} + {6}.00{\text{BC}}{-}{ 1}.{\text{13A}}^{{2}} + { 25}.{\text{87B}}^{{2}} {-}{ 37}.{\text{37C}}^{{2}} \hfill \\ \end{gathered} $$

9 $$ \begin{aligned} {\text{Biogas production for Actual Factors }} & = {-}{ 33}.00 \, + { 13}0.{\text{25 Biomass amount }}{-}{ 24}.{\text{37Co}}_{{3}} {\text{O}}_{{4}} {\text{NPs dose}}  \\  & \quad + { 19}.0{\text{9 Contact Time}}{-}{ 6}.{\text{9 Biomass amount }} \times {\text{Co}}_{{3}} {\text{O}}_{{4}} {\text{NPs dose}}  \\ & \quad + { 1}.{\text{45 Biomass amount }} \times {\text{ Contact Time }} + \, 0.{\text{11Co}}_{{3}} {\text{O}}_{{4}} {\text{NPs dose}} \\ &\quad\times {\text{ Contact Time}}{-}{ 4}.{\text{5 Biomass amount}}^{{2}} {-}{ 1}.0{\text{4Co}}_{{3}} {\text{O}}_{{4}} {\text{NPs dose}}^{{2}} \hfill \\ & \quad  {-} \, 0.{\text{31 Contact Time}}^{{2}} \hfill \\ \end{aligned} $$

10 $$ {\text{Biomethane production for Coded Factors }} = {3}0.{26} + {6}.{\text{94A}}{-}{ 8}.{\text{65B}} + { 13}.{\text{88C}} + { 1}.{\text{61AB}} + { 5}.{\text{12AC }}{-}0.0{\text{4BC}}{-}{ 1}.{\text{28A}}^{{2}} + { 13}.{\text{22B}}^{{2}} {-}{ 1}0.{\text{36C}}^{{2}} $$

11 $$ \begin{gathered} {\text{Biomethane production for Actual Factors }} = {38}.{98 }{-}{ 2}.{\text{77 Biomass amount}}{-}{ 12}.{\text{93Co}}_{{3}} {\text{O}}_{{4}} {\text{NPs dose}} + {4}.{1}0{\text{ Contact Time}}0.{\text{644 Biomass amount}} \hfill \\ \, \times {\text{Co}}_{{3}} {\text{O}}_{{4}} {\text{NPs dose}} + 0.{\text{931 Biomass amount }} \times {\text{ Contact Time }}{-} \, 0.000{\text{7Co}}_{{3}} {\text{O}}_{{4}} {\text{NPs dose}} \times {\text{ Contact Time}}{-}{ 5}.{\text{14 Biomass amount}}^{{2}} \hfill \\ {-}0.{\text{529Co}}_{{3}} {\text{O}}_{{4}} {\text{NPs dose}}^{{2}} {-}0.0{\text{86 Contact Time}}^{{2}} \hfill \\ \end{gathered} $$

**Table 6:** Co_3_O_4_NPs are being used in an experimental design to produce biogas and biomethane.

Run	Independent factors			Biogas generation		CH_4_ generation	
	Biomass amount (A)	Co_3_O_4_NPs dose (B)	Contact time (C)	Exp	Predicted	Exp	Predicted
1	0.5	15	22	156	186	15.28	24.99
2	1	5	33	333	347.38	47.11	55.69
3	1	15	11	86	71.63	19.21	10.63
4	1	10	22	227	227	30.26	30.26
5	1.5	10	11	105	125.63	2.35	6.56
6	1	10	22	227	227	30.26	30.26
7	0.5	10	11	73	57.37	4.05	2.92
8	1.5	10	33	320	335.63	43.43	44.57
9	1	10	22	227	227	30.26	30.26
10	1.5	15	22	242	235.75	37.73	42.1
11	1	10	22	227	227	30.26	30.26
12	0.5	5	22	227	233.25	49.87	45.5
13	1.5	5	22	382	352	65.88	56.17
14	1	15	33	287	277.63	43.82	38.31
15	0.5	10	33	256	235.37	24.64	20.43
16	1	5	11	156	165.37	22.34	27.84
17	1	10	22	227	227	30.26	30.26

The equation written in terms of the basic components allows one to predict the reaction for certain concentrations of each element. Here, the levels of each component must be stated in terms of their original units. Since the coefficients are scaled to take into account the units of each element and the intercept is not at the centre of the design space, this equation should not be used to establish the relative value of each factor.

**Figure 13:**
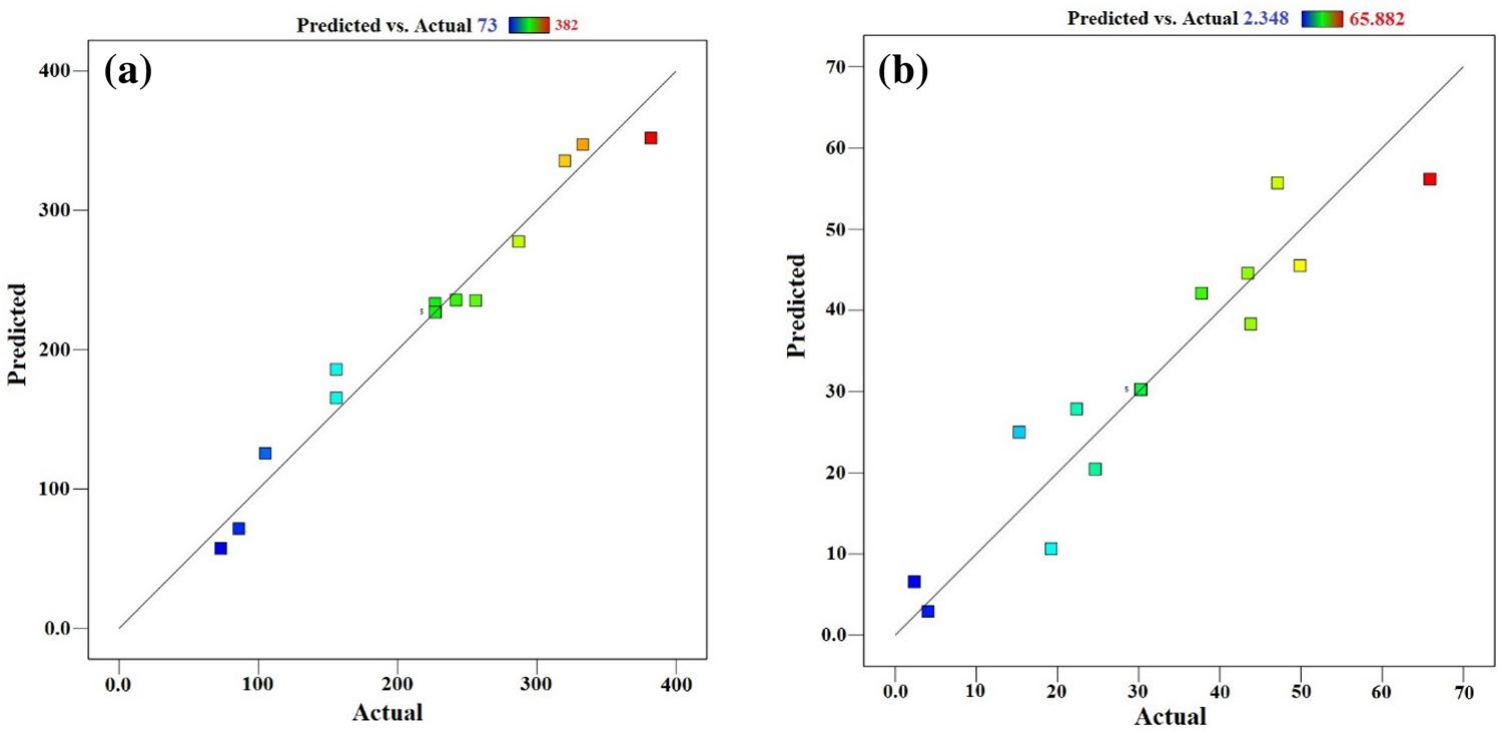
Correlation between the predicted and actual results for (**a**) biogas (**b**) biomethane production (mL/g VS).

In Fig. 13, a link between anticipated and actual biogas and methane generation from algae is depicted. The image clearly illustrates the substantial agreement between experimental results and the model predictions, which is reinforced by the high *R*^2^ values (*R*^2^ = 0.967 and 0.889 for biogas and biomethane, respectively). Tables 7 and 8 include the results of the ANOVA for the various Co_3_O_4_NPs concentrations under investigation. This analysis is used to forecast the independent variables' cubic, individual, and interaction impacts on the generation of biogas and biomethane. The findings indicate that for all treatments, except for quadratic, which has a higher *R*^2^, both the quadratic and linear models (*P*-value < 0.05) have a substantial influence. The adjusted *R*^2^ and the expected *R*^2^ accord rather well. Adeq Precision does signal-to-noise ratio (S/N) measurement. A ratio of at least 4 is preferred. The S/N values for biomethane and biogas, respectively, of 8.45 and 16.47, reveal sufficient and strong RSM model signals, which may be applied to guide the design^56–59^.

**Table 7:** ANOVA and a summary of model fit for the Box-Behnken design for producing biogas.

Source	Value	Sum of squares	Df	Mean square	F-value	p-value	Remarks	source	SD	R^2^	Adjusted R^2^	Predicted R^2^	PRESS	Remarks
Model	–	1.127E+05	9	12,525.85	23.04	0.0002	Significant	–	–	–	–	–	–	–
A-biomass amount	–	14,196.13	1	14,196.13	26.11	0.0014		–	–	–	–	–	–	–
B-Co_3_O_4_NPs dose	–	13,366.12	1	13,366.12	24.58	0.0016		–	–	–	–	–	–	–
C-contact time	–	75,272.00	1	75,272.00	138.43	< 0.0001		–	–	–	–	–	–	–
AB	–	1190.25	1	1190.25	2.19	0.1825		–	–	–	–	–	–	–
AC	–	256.00	1	256.00	0.4708	0.5147		–	–	–	–	–	–	–
BC	–	144.00	1	144.00	0.2648	0.6227		–	–	–	–	–	–	–
A^2^		5.33	1	5.33	0.0098	0.9239		–	–	–	–	–	–	–
B^2^		2819.01	1	2819.01	5.18	0.0569		–	–	–	–	–	–	–
C^2^		5881.64	1	5881.64	10.82	0.0133		–	–	–	–	–	–	–
Residual	–	3806.25	7	543.75				–	–	–	–	–	–	–
Lack of fit	–	3806.25	3	1268.75				–	–	–	–	–	–	–
Pure error	–	0.0000	4	0.0000				–	–	–	–	–	–	–
Cor total	–	1.165E+05	16					–	–	–	–	–	–	–
SD	23.32	–		–	–	–	–	–	–	–	–	–	–	–
Mean	221.06	–		–	–	–	–	–	–	–	–	–	–	–
C.V.%	10.55	–		–	–	–	–	–	–	–	–	–	–	–
R^2^	0.9673	–	–	–	–	–	–	–	–	–	–	–	–	–
Adjusted R^2^	0.9253	–	–	–	–	–	–	–	–	–	–	–	–	–
Predicted R^2^	0.4774	–	–	–	–	–	–	–	–	–	–	–	–	–
Adeq precision	16.4738	–	–	–	–	–	–	–	–	–	–	–	–	–
Linear	–	–	–	–	–	–	–	–	32.47	0.8824	0.8553	0.7553	28,517.22	Suggested
2FI	–	–	–	–	–	–	–	–	34.81	0.8960	0.8337	0.4720	61,533.79	
Quadratic	–	–	–	–	–	–	–	–	23.32	0.9673	0.9253	0.4774	60,900.00	Suggested
Cubic	–	–	–	–	–	–	–	–	0.0000	1.0000	1.0000		*	Aliased

*Case(s) with leverage of 1.0000: PRESS statistic not define.

**Table 8:** ANOVA and a summary of model fit for the Box-Behnken design for producing biomethane.

Source	Value	Sum of Squares	df	Mean Square	F-value	p-value	Remarks	source	SD	R^2^	Adjusted R^2^	Predicted R^2^	PRESS	Remarks
Model	–	3775.54	9	419.50	6.21	0.0125	significant	–	–	–	–	–	–	–
A-biomass amount	–	385.68	1	385.68	5.71	0.0482		–	–	–	–	–	–	–
B-Co_3_O_4_NPs dose	–	598.10	1	598.10	8.86	0.0206		–	–	–	–	–	–	–
C-contact time	–	1541.60	1	1541.60	22.84	0.0020		–	–	–	–	–	–	–
AB	–	10.37	1	10.37	0.1536	0.7068		–	–	–	–	–	–	–
AC	–	104.99	1	104.99	1.56	0.2525		–	–	–	–	–	–	–
BC	–	0.0064	1	0.0064	0.0001	0.9925		–	–	–	–	–	–	–
A^2^		6.94	1	6.94	0.1028	0.7578		–	–	–	–	–	–	–
B^2^		735.44	1	735.44	10.89	0.0131		–	–	–	–	–	–	–
C^2^		451.53	1	451.53	6.69	0.0361		–	–	–	–	–	–	–
Residual	–	472.55	7	67.51				–	–	–	–	–	–	–
Lack of fit	–	472.55	3	157.52				–	–	–	–	–	–	–
Pure error	–	0.0000	4	0.0000				–	–	–	–	–	–	–
Cor total	–	4248.09	16					–	–	–	–	–	–	–
SD	8.22	–		–	–	–	–	–	–	–	–	–	–	–
Mean	31.00	–		–	–	–	–	–	–	–	–	–	–	–
C.V.%	26.50	–		–	–	–	–	–	–	–	–	–	–	–
R^2^	0.8888	–	–	–	–	–	–	–	–	–	–	–	–	–
Adjusted R^2^	0.7457	–	–	–	–	–	–	–	–	–	–	–	–	–
Predicted R^2^	–0.7798	–	–	–	–	–	–	–	–	–	–	–	–	–
Adeq precision	8.4508	–	–	–	–	–	–	–	–	–	–	–	–	–
Linear	–	–	–	–	–	–	–	–	11.51	0.5945	0.5009	0.1518	3603.43	Suggested
2FI	–	–	–	–	–	–	–	–	12.68	0.6216	0.3946	–0.9414	8247.19	
Quadratic	–	–	–	–	–	–	–	–	8.22	0.8888	0.7457	–0.7798	7560.83	Suggested
Cubic	–	–	–	–	–	–	–	–	0.0000	1.0000	1.0000		*	Aliased

*Case(s) with leverage of 1.0000: PRESS statistic not defined.

#### Simultaneous effects of interactive variables

The effects and interactions of the independent variables Biomass quantity, Co_3_O_4_NPs dosage, and Contact Time on the generation of biogas and biomethane are shown in three-dimensional surface plots.

**Figure 14:**
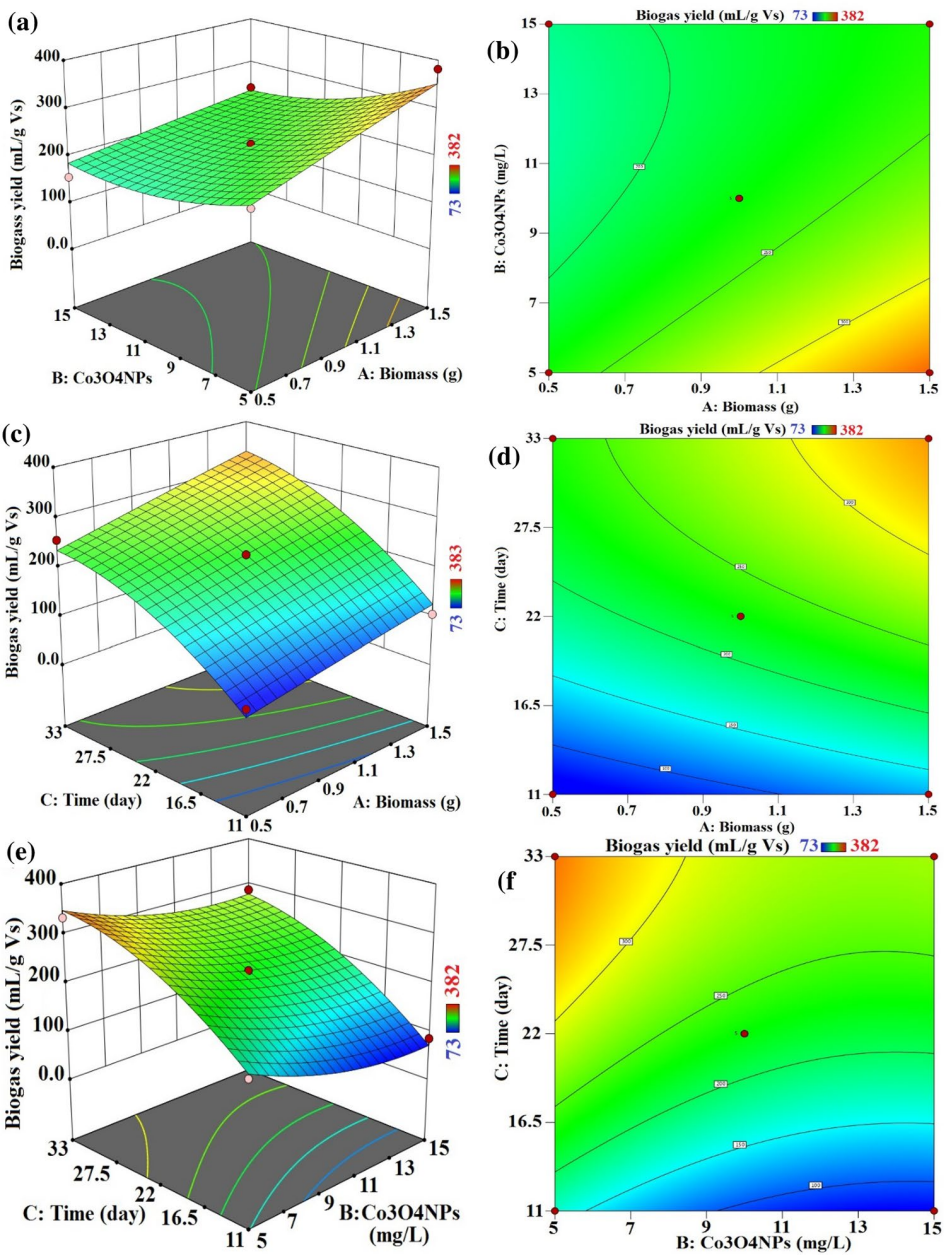
Process variables combined influence. (**a**,**b**) Biomass amount and Co_3_O_4_NPs dose, (**c**,**d**) biomass amount and contact time and (**e**,**f**) Co_3_O_4_NPs dose and contact time on biogas production (mL/g VS) with interaction influence of dual factors.

**Figure 15:**
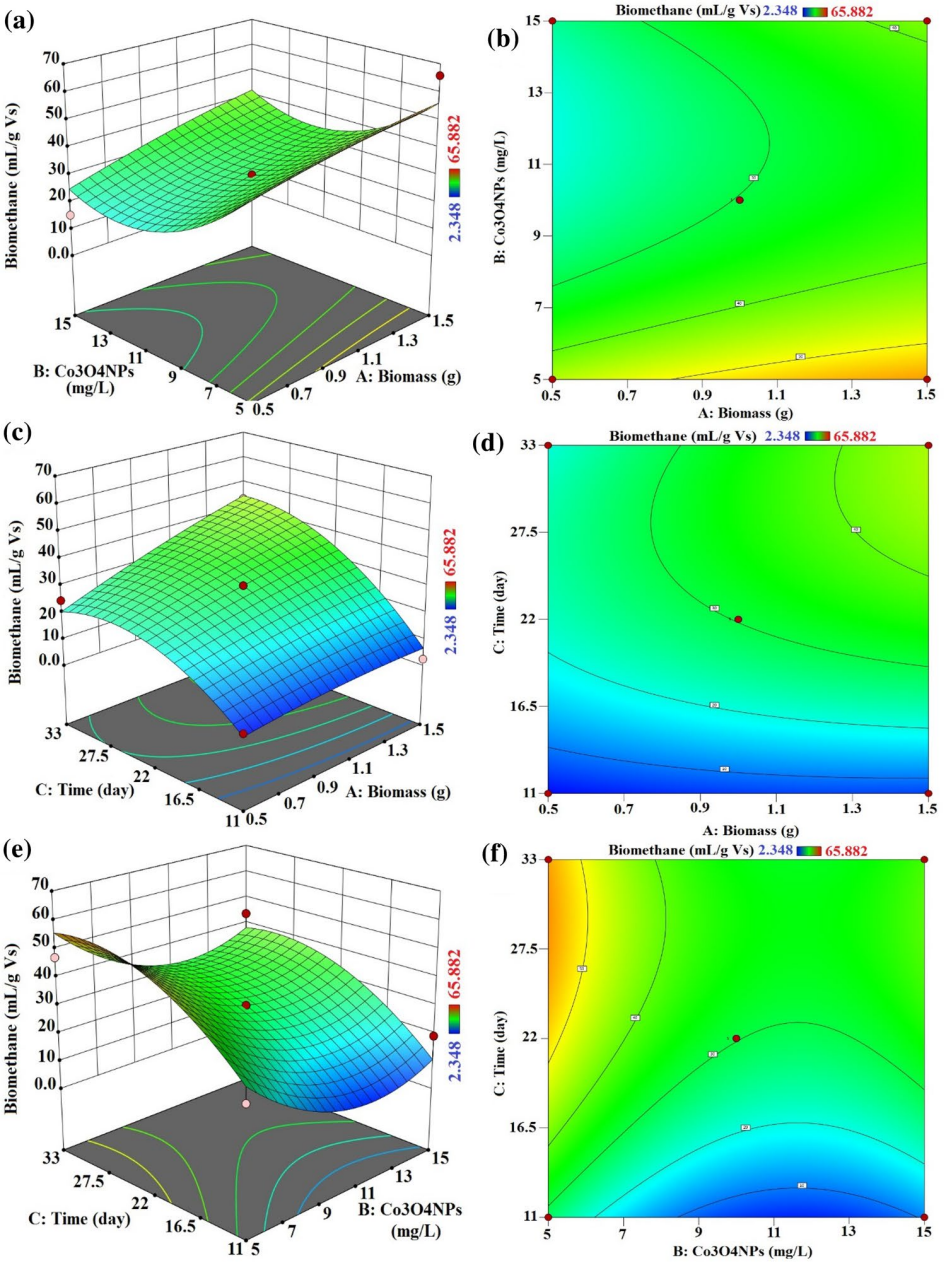
Process variables combined influence (**a**,**b**) Biomass amount and Co_3_O_4_NPs dose, (**c**,**d**) Biomass amount and contact time and (**e**,**f**) Co_3_O_4_NPs dose and contact time on biomethane production (mL/g VS) with interaction influence of dual factors.

The interaction between the quantity of biomass and the dosage of Co_3_O_4_NPs, as illustrated in Figs. 14 and 15, demonstrate the major impact of both parameters on biogas and biomethane production. The biogas production increased with increasing Co_3_O_4_NPs for a lower and higher dosage of biomass (0.5 and 1.0 g). Alternatively, in the case of biomethane production, the Co_3_O_4_NPs has an inhibitory effect on the biomethane production in all treatments except in the case of lower biomass amount and lower dosage of Co_3_O_4_NPs 5 mg/L, which has result same as the control sample. These data established that the biogas production rate was very dependent on the amount of Co_3_O_4_NPs and also on the biomass amount. Moreover, the amount of biomethane mainly depends on the catalyst dosage, and the increase of biogas does not mainly the increase of biomethane due to the inhibitory effect of Co_3_O_4_NPs on the methanogenic bacteria.

**Figure 16:**
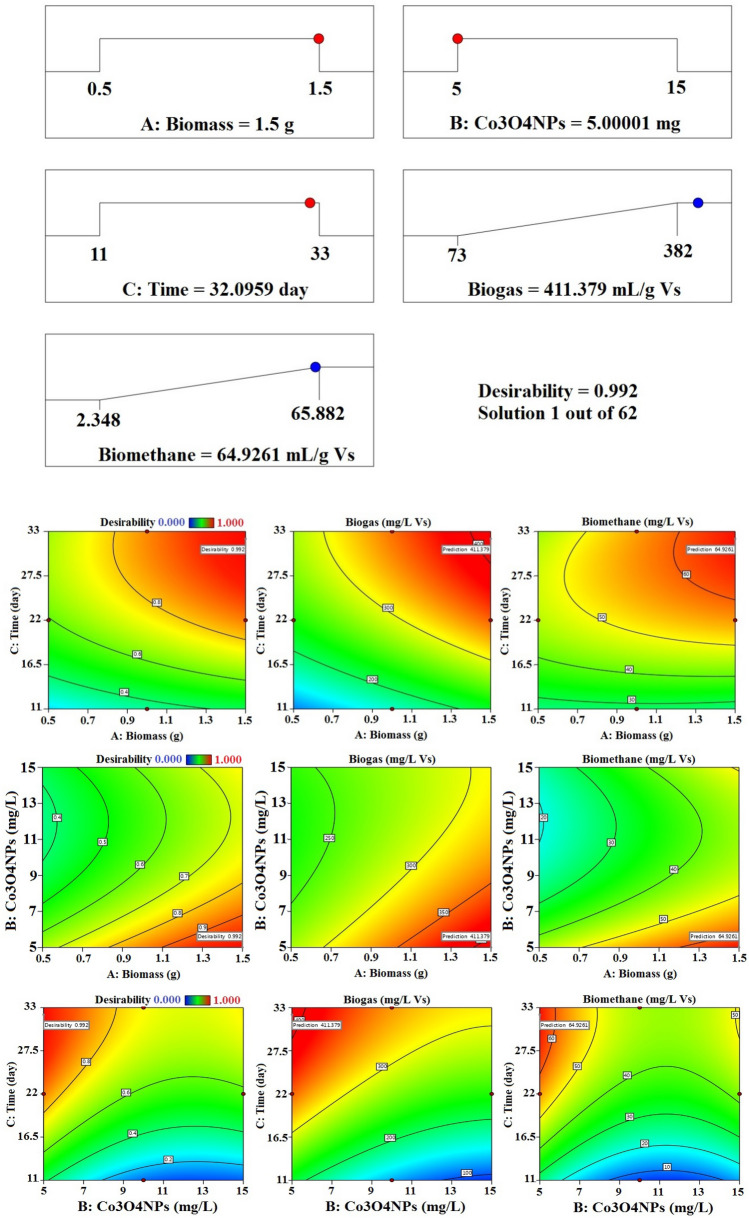
Optimum conditions predicted by RSM method for biogas and biomethane production (mL/g VS).

An additional statistical design calculation was performed under the same experimental conditions in order to optimise and validate the proposed mathematical model. The results of the mathematical model indicate that the higher desirability value is equal to 0.992, as seen in Fig. 16. The maximal biogas and biomethane output for the experiment under these circumstances was 411.38 (mL/g VS) and 64.93 (mL/g VS), respectively. This was accomplished for treatment with 5 mg/L of Co_3_O_4_NPs, corresponding to the contact time of 32 days, 1.5 g of biomass (Fig. 15a).

### Techno-economic analysis of Co_3_O_4_NPs

To ascertain the effect of Co_3_O_4_ NPs on the generation of biogas, a techno-economic study was carried out (Table 9). Based on the amount of biogas produced from 1.0 m^3^ of substrate in contrast to manure, which has a net profit of $0.00 USD, the cost and net energy are assessed. Co_3_O_4_ NPs at a concentration of 5 mg/L provided the most net energy (Table 9). This led to a 0.60 USD net profit. However, when 10 mg/L of Co_3_O_4_NPs was employed, the lowest amount of net energy that could be extracted from biogas was 1.95 kWh, and the net profit was 0.42 USD. In addition to the techno-economic advantages of using Co_3_O_4_ NPs in energy generation, biogas fertilizer is an alternative fertilizer that, when compared to traditional chemical fertilizers, may provide a number of environmental advantages that need further study.

**Table 9:** Techno-economic analysis of Co_3_O_4_-NPs addition for biogas production.

Calculation	Energy balance/m^3^ of substrate		Cost and income ($)			
	Cumulative biogas production (CBP)*	Biogas energy content (BEC)** (kWh/m^3^)	Energy cost (C_X_)** (USD/kWh)	NPs chemicals and preparation cost (USD)	Net income (C_Z_) (USD)	Net profit (USD) (C_Z_-Control C_Z_)
Reference (manure)*	0.05	0.305	0.0705	0	0.0705	0
Co_3_O_4_-NPs (5 mg/L)	0.46	2.82	0.65	− 0.02	0.67	0.60
Co_3_O_4_-NPs (10 mg/L)	0.32	1.95	0.45	− 0.04	0.49	0.42
Co_3_O_4_-NPs (15 mg/L)	0.36	2.17	0.50	− 0.06	0.56	0.49

*The reference for CBP of manure was taken from manure reading in this experiment.

**Values for BEC, Cx, Cy were taken after Ali et al.,^25^.

## Conclusion

Co_3_O_4_NPs were used to investigate its impact on biogas and biomethane production from green algae *C. myrica* as a source of biomass using BBD. The structure of the fabricated Co_3_O_4_NPs was confirmed using FTIR, SEM, TEM and XRD. The data suggested that adding 5 mg/L Co_3_O_4_NPs to *C. myrica* (1.5 g) significantly increased biogas generation (462 mL/g VS). The maximum biomethane generation (61.4 mL/g VS) was acquired with a control sample *of C. myrica* (1.5 g). The data demonstrated that the best conditions for the supreme biogas and biomethane production for all experiments 411.38 (mL/g VS) and 64.93 (mL/g VS), respectively, were attained for 5 mg/L of Co_3_O_4_NPs treatment corresponds to the contact time: 32 days, biomass amount 1.5 g. The results concluded that the impact of Co_3_O_4_NPs on purity and amount of biogas mainly depends on the following: (1) the type and the nature of the CoNPs where it is Co, or CoONPs, Co_3_O_4_, besides the dosage optimization. (2) Depending on the C/N ratio the substrate type and amount may need to be optimized. The higher amount of Co_3_O_4_ nanoparticles may form aggregates, act as electron traps, and hinder DIET. ROS can play a positive role in biogas production by stimulating microbial activity and electron transfer, while excessive ROS production can reduce biogas and methane production.

## Data Availability

The corresponding author of the study can provide access to the datasets utilized in this inquiry upon request.
